# Colistin exerts potent activity against *mcr^+^* Enterobacteriaceae via synergistic interactions with the host defense

**DOI:** 10.1172/JCI170690

**Published:** 2025-04-22

**Authors:** Monika Kumaraswamy, Angelica Montenegro Riestra, Anabel Flores, Samira Dahesh, Fatemeh Askarian, Satoshi Uchiyama, Jonathan Monk, Sean Jung, Gunnar Bondsäter, Victoria Nilsson, Melanie Chang, Jüergen B. Bulitta, Yinzhi Lang, Armin Kousha, Elisabet Bjånes, Natalie Chavarria, Ty’Tianna Clark, Hideya Seo, George Sakoulas, Victor Nizet

**Affiliations:** 1 Division of Infectious Diseases, Department of Medicine, and; 2 Department of Cellular and Molecular Biology, University of Texas at Tyler, Tyler, Texas, USA.; 3 Division of Infectious Diseases and Global Public Health, Department of Medicine, University of California, San Diego (UCSD), La Jolla, California, USA.; 4 Infectious Diseases Section, VA San Diego Healthcare System, San Diego, California, USA.; 5 Department of Biology, San Diego State University, San Diego, California, USA.; 6 Division of Host-Microbe Systems and Therapeutics, Department of Pediatrics, UCSD, La Jolla, California, USA.; 7 Department of Biological Sciences, California Baptist University, Riverside, California, USA.; 8 Department of Bioengineering, UCSD, La Jolla, California, USA.; 9 Faculty of Medicine, Lund University, Lund, Sweden.; 10 School of Medicine, China Medical University, Taichung, Taiwan.; 11 Department of Pharmacotherapy and Translational Research, University of Florida, Gainesville, Florida, USA.; 12 Department of Anesthesia, Kyoto University, Kyoto, Japan.; 13 Sharp Rees Stealy Medical Group, San Diego, California, USA.; 14 Skaggs School of Pharmacy and Pharmaceutical Sciences, UCSD, La Jolla, California, USA.

**Keywords:** Infectious disease, Microbiology, Bacterial infections, Drug therapy

## Abstract

Colistin (COL) is a cationic cyclic peptide that disrupts the membranes of Gram-negative bacteria and is often used as a last resort antibiotic against multidrug-resistant strains. The emergence of plasmid-borne *mcr* genes, which confer transferable COL resistance, has raised serious concerns, particularly in strains also carrying extended-spectrum β-lactamase and carbapenemase genes. Standard antimicrobial susceptibility testing (AST), performed in enriched bacteriological media, indicates no activity of COL against *mcr*^+^ strains, leading to its exclusion from treatment regimens. However, these media poorly reflect in vivo physiology and lack host immune components. Here we show that COL retained bactericidal activity against *mcr-1*^+^ *Escherichia coli*, *Klebsiella pneumoniae*, and *Salmonella enterica* when tested in tissue culture medium containing physiological bicarbonate. COL enhanced serum complement deposition on bacterial surfaces and synergized with human serum to kill pathogens. At clinically achievable concentrations, COL killed *mcr-1*^+^ strains in freshly isolated human blood and was effective as monotherapy in a murine *E*. *coli* bacteremia model. These findings suggest that COL, currently dismissed based on conventional AST, may offer clinical benefit against *mcr-1*^+^ infections when evaluated under more physiological conditions — warranting reconsideration in clinical microbiology practices and future trials for high-risk patients.

## Abstract 2

Last line antibiotic colistin shows hidden activity against presumed multidrug-resistant mcr+ Gram-negative bacterial pathogens revealed only in the presence of physiologic media or host immunity.

## Graphical abstract

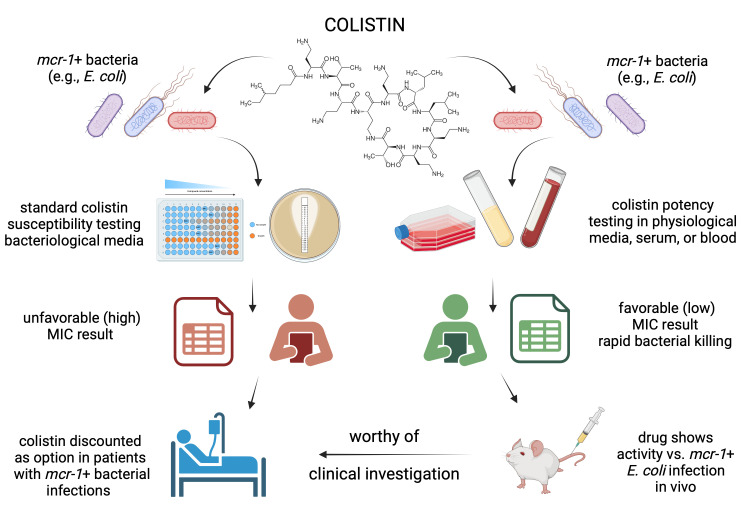

## Introduction

The non-ribosomally synthesized cationic polypeptide antibiotic colistin (COL; polymyxin E) was first isolated from the soil bacterium *Paenibacillus polymyxa* subsp. *colistinus* in 1949 (1). COL has dose-dependent bactericidal activity against most Gram-negative bacteria, but its usage was largely abandoned in the 1970s because of associated nephro- and neurotoxicity and the emergence of less toxic antibiotic alternatives such as cephalosporins, aminoglycosides, and quinolones. However, an increasing prevalence of multidrug-resistant (MDR) Gram-negative infections caused by carbapenem-resistant Enterobacteriaceae (CRE), *Acinetobacter baumannii*, and *Pseudomonas aeruginosa* with limited therapeutic options has prompted a resurgence of COL usage as a last line of defense (2, 3) and its designation by the World Health Organization as a critically important antimicrobial for human medicine (4). As COL resistance determinants emerge in CRE and other highly MDR Gram-negative bacteria (5–8), a prospect of strains resistant to all conventional antibiotics is widely feared.

A multicomponent polypeptide composed of a hydrophilic cyclic heptapeptide, a tripeptide side chain, and a hydrophobic acylated fatty acid residue (6-methyl-octanoic acid or 6-methyl-heptanoic acid), COL binds to lipopolysaccharides (LPSs) on the outer membrane of target Gram-negative bacteria (9). Like other polymyxins, COL contains cationic α,γ-diaminobutyric acid residues that competitively displace LPS-stabilizing divalent cations (Mg^2+^ and Ca^2+^) from negatively charged phosphate groups, leading to cell envelope permeabilization, leakage of intracellular contents, and bacterial cell death (9). The primary mechanisms of bacterial resistance to COL involve covalent cationic modifications to LPS structure, including the addition of phosphoethanolamine, 4-amino-4-deoxy-l-arabinose, and/or galactosamine to the phosphate group of lipid A or core oligosaccharide (10). These alterations neutralize LPS negative charge, thereby diminishing COL binding affinity to its target (9).

Historically, genetic determinants of COL resistance were localized to the chromosome, impeding widespread dissemination through and across bacterial populations and limiting clinical impact (10). This changed dramatically upon the emergence of the horizontally transferable plasmid-mediated mobilized colistin resistance (*mcr-1*) determinant encoding an LPS-modifying transferase enzyme that adds phosphoethanolamine to lipid A. First identified in 2015 among *Escherichia coli* strains in pigs and humans in China (11), *mcr-1* has since been found in a wide range of environmental, animal, and human clinical isolates worldwide, representing a significant global public health threat (12, 13). Furthermore, numerous studies have documented diversification of a large array of plasmid-borne *mcr* variants, from *mcr*-*2* to *mcr*-*10* (14–20), highlighting the success of this resistance paradigm to evolutionary selective forces. Spread of *mcr-1* and variants now encompasses Gram-negative bacteria harboring extended-spectrum β-lactamase and carbapenemase resistance genes, threatening the therapeutic utility of our full existing antibiotic armamentarium (5–8).

COL is never recommended in the therapy of Gram-negative bacteria upon molecular detection of *mcr-1* or related plasmids. This medical decision-making reflects consistent elevated minimum inhibitory concentration (MIC) values identified by antimicrobial susceptibility testing (AST) in the clinical microbiology laboratory. AST is routinely performed using a nutrient-rich bacteriological medium, specifically cation-adjusted Mueller-Hinton broth (CA-MHB), with standardized protocols and breakpoints developed and regularly updated by the Clinical and Laboratory Standards Institute (CLSI) in the United States (21) and the European Committee on Antimicrobial Susceptibility Testing (EUCAST) (22). However, when faced with MDR bacterial isolates exhibiting few if any drug susceptibilities and a high incidence of clinical treatment failure, we (23–25) and others (26–28) have advocated greater circumspection regarding key limitations of allowing a single “gold standard” in vitro AST assay to guide antibiotic selection. First, AST is performed in medium (CA-MHB) composed of beef extract, casein, and starch, chosen for optimal bacterial growth — a molecular composition entirely distinct from that of infected human tissues and fluids where the bacterial targets of antibacterial action are located. Second, AST incorporates no molecular or cellular elements of host innate immunity such as endogenous antimicrobial peptides, serum complement, or phagocytes, which may interact with any given pharmacological antibiotic synergistically or antagonistically. Recent studies have shown that diverse drugs including ampicillin (29), nafcillin (30), azithromycin (24, 31), rifabutin (32), and avibactam (33), to which MDR strains of key human bacterial pathogens are deemed highly resistant by standard MIC testing, do indeed exhibit potent (but neglected) antimicrobial activities against these same strains when AST is performed in medium that closely mimics human physiology or in synergy with host immune factors.

Given the urgent concern of plasmid-borne COL resistance now spreading within the highest threat MDR Gram-negative bacterial pathogens, we chose to investigate the impact of more physiological medium conditions and host defense components on the susceptibility of various medically important Enterobacteriaceae harboring *mcr* gene variants (*mcr-1* to *mcr-4*) to COL and the related cationic peptide antibiotic polymyxin B (PMB). Our in vitro, ex vivo, and in vivo studies in this context uncover clear, and likely therapeutically meaningful, COL and PMB activities against diverse *mcr^+^* Gram-negative bacterial pathogens. These activities are currently hidden from practitioners who remain reliant on standard AST to guide management of critically ill patients, a matter that we propose deserves careful attention and further clinical investigation.

## Results

### Polymyxins retain activity against mcr^+^ Gram-negative bacteria in more physiological medium testing conditions.

We compared the MICs of COL and PMB against 12 strains of Gram-negative bacteria (7 *E*. *coli*, 4 *Salmonella* spp., 1 *Klebsiella pneumoniae*), each harboring plasmid-mediated colistin resistance (*mcr-1* to *mcr-4*) genes, in (a) standard bacteriological testing medium (CA-MHB) versus (b) a more physiological medium based on the common mammalian tissue culture medium Roswell Park Memorial Institute (RPMI) 1640 (34). To ensure equivalent bacterial growth kinetics, the RPMI 1640 medium was supplemented with 10% Luria-Bertani broth and designated RPMI(10%LB) (24). A strong multifold reduction in COL and PMB MICs was observed for all isolates tested in RPMI(10%LB) compared with CA-MHB, with the COL MIC dropping to ≤2 mg/L for 12 of 12 strains and the PMB MIC dropping to ≤2 mg/L for 11 of 12 strains in the more physiological medium (Table 1). Notably, the current clinical MIC breakpoint of COL established by EUCAST for Enterobacterales is ≤2 mg/L (22), a concentration readily achievable in serum by standard therapeutic dosing of intravenous (i.v.) colistimethate sodium (35).

### COL activity against mcr-1^+^ Gram-negative bacteria in physiological medium is bactericidal and dependent on bicarbonate and involves membrane permeabilization.

**Figure 1:**
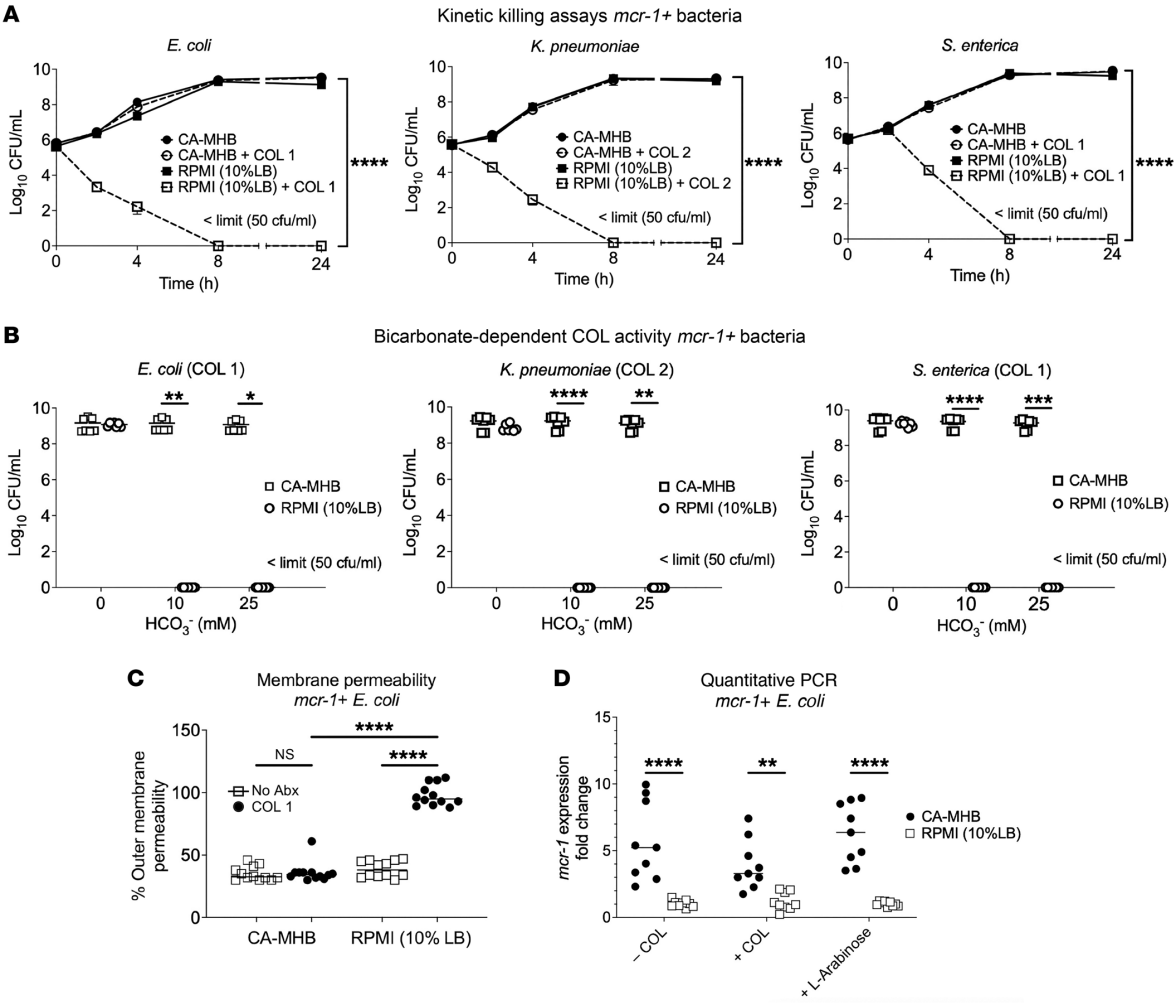
COL bactericidal activity against *mcr-1^+^* Gram-negative pathogens in physiological medium is dependent on bicarbonate and involves membrane permeabilization. (**A**) Kinetic kill curves show COL minimum bactericidal concentration against *E*. *coli*, *K*. *pneumoniae*, and *S*. *enterica* (defined as a reduction in viable bacteria ≥3 log_10_ CFU/mL at 24 hours vs. starting inoculum) when assessed in supplemented mammalian tissue culture RPMI(10%LB) versus standard bacteriological medium CA-MHB; limit of detection <50 CFU/mL. (**B**) Bactericidal activity of COL in RPMI(10%LB) or CA-MHB medium amended with 0, 10, or 25 mM HCO_3_^–^; limit of detection <50 CFU/mL. (**C**) COL-mediated outer membrane permeabilization of *mcr-1^+^* *E*. *coli* assessed using the nonpolar compound 1-*N*-phenylnaphthylamine (NPN) that fluoresces strongly in phospholipid environments in RPMI(10%LB) or CA-MHB. (**D**) Relative bacterial surface charge estimated by cationic cytochrome *c* binding to *mcr-1^+^* *E*. *coli* following growth in CA-MHB or RPMI(10%LB). Data represent the mean ± SEM from the combination of 3 experiments performed in triplicate. *P ≤ 0.05, **P ≤ 0.01, ***P ≤ 0.001, ****P ≤ 0.0001.

Medium-dependent COL susceptibility was further examined in kinetic killing assays performed with *mcr*-*1*–harboring strains of *E*. *coli*, *K*. *pneumoniae*, and *Salmonella* *enterica*, each at an initial inoculum of 5 × 10^5^ CFU/mL. COL at 1 or 2 mg/L showed potent bactericidal activity against all 3 bacterial strains in the more physiological RPMI(10%LB) medium, with no recovered bacteria after 8 hours (Figure 1A). In stark contrast, the same assay performed in standard bacteriological medium CA-MHB found that all three *mcr-1^+^* Gram-negative bacteria achieved rapid logarithmic growth despite the addition of 1 or 2 mg/L COL (Figure 1A). We further conducted kinetic killing assays to assess the activity of COL and PMB against a wider spectrum of *mcr^+^* variants in Gram-negative bacterial isolates (*mcr-1* to *mcr-4*) in both CA-MHB and RPMI(10%LB) (Supplemental Figure 1; supplemental material available online with this article; https://doi.org/10.1172/JCI170690DS1). Notably, bactericidal activity of COL and PMB against *mcr-1^+^*, *mcr-2^+^*, *mcr-3^+^*, and *mcr-4^+^* *E*. *coli* or *S*. *enterica* was consistently observed in RPMI(10%LB) via time kill curves.

Bicarbonate (HCO_3_^–^), the major buffering anion for maintenance of mammalian physiological pH, is a key constituent of RPMI 1640 and other tissue culture media that is lacking in CA-MHB. The presence of HCO_3_^–^ can influence MIC results, sometimes increasing antibiotic potency, e.g., for macrolides or aminoglycosides against several Gram-negative bacteria (24, 36), and other times decreasing antibiotic potency, e.g., for tetracyclines against similar bacterial species (36). We repeated our kinetic killing studies of the *mcr*-*1^+^* *E*. *coli*, *K*. *pneumoniae*, and *S*. *enterica* strains in bicarbonate-free RPMI(10%LB) into which we titrated NaHCO_3_^–^ to achieve lower (10 mM) and higher (25 mM) final concentrations spanning a range characteristic of in vivo conditions. Whereas complete bactericidal activity of 1 or 2 mg/L COL against all 3 species of *mcr-1^+^* bacteria was seen in the presence of low or high HCO_3_^–^, activity was completely lost in the absence of the anion (Figure 1B). Although essential for sensitizing *mcr-1^+^* bacteria to COL killing in physiological medium, HCO_3_^–^ was insufficient to sensitize the same bacteria to COL in the CA-MHB bacteriological medium (Figure 1B).

Changes in *E*. *coli* outer membrane permeabilization upon COL exposure were estimated using the small hydrophobic molecule 1-*N*-phenylnaphthylamine (NPN), which fluoresces weakly in aqueous environments but strongly when membrane integrity is compromised, binding to bacterial membrane phospholipids after entering the periplasmic space (37). COL treatment of *mcr-1^+^* *E*. *coli* markedly increased NPN fluorescence (outer membrane permeabilization) in RPMI(10%LB), consistent with its observed bactericidal activity in this medium, but produced no distinguishable change from baseline fluorescence when tested in CA-MHB (Figure 1C). To determine whether the increased susceptibility and outer membrane permeabilization of *mcr-1^+^* *E*. *coli* to COL in RPMI(10%LB) compared with CA-MHB were attributable to reduced *mcr-1* expression, we performed quantitative real-time PCR (RT-PCR) using *E*. *coli* cultured in RPMI(10%LB) or CA-MHB, in both the absence and presence of subtherapeutic COL (0.25 μg/mL) or 0.02% l-arabinose — a potent inducer of *mcr-1* expression (Figure 1D). In each case, *mcr-1* transcript levels were multifold lower when the *E*. *coli* strain was grown in RPMI(10%LB) compared with CA-MHB, suggesting that reduced *mcr-1* expression may underlie *E*. *coli* hypersusceptibility to COL and NPN outer membrane permeabilization in host-mimicking media.

### COL accelerates killing of mcr-1^+^ Gram-negative bacteria in human blood and serum.

**Figure 2:**
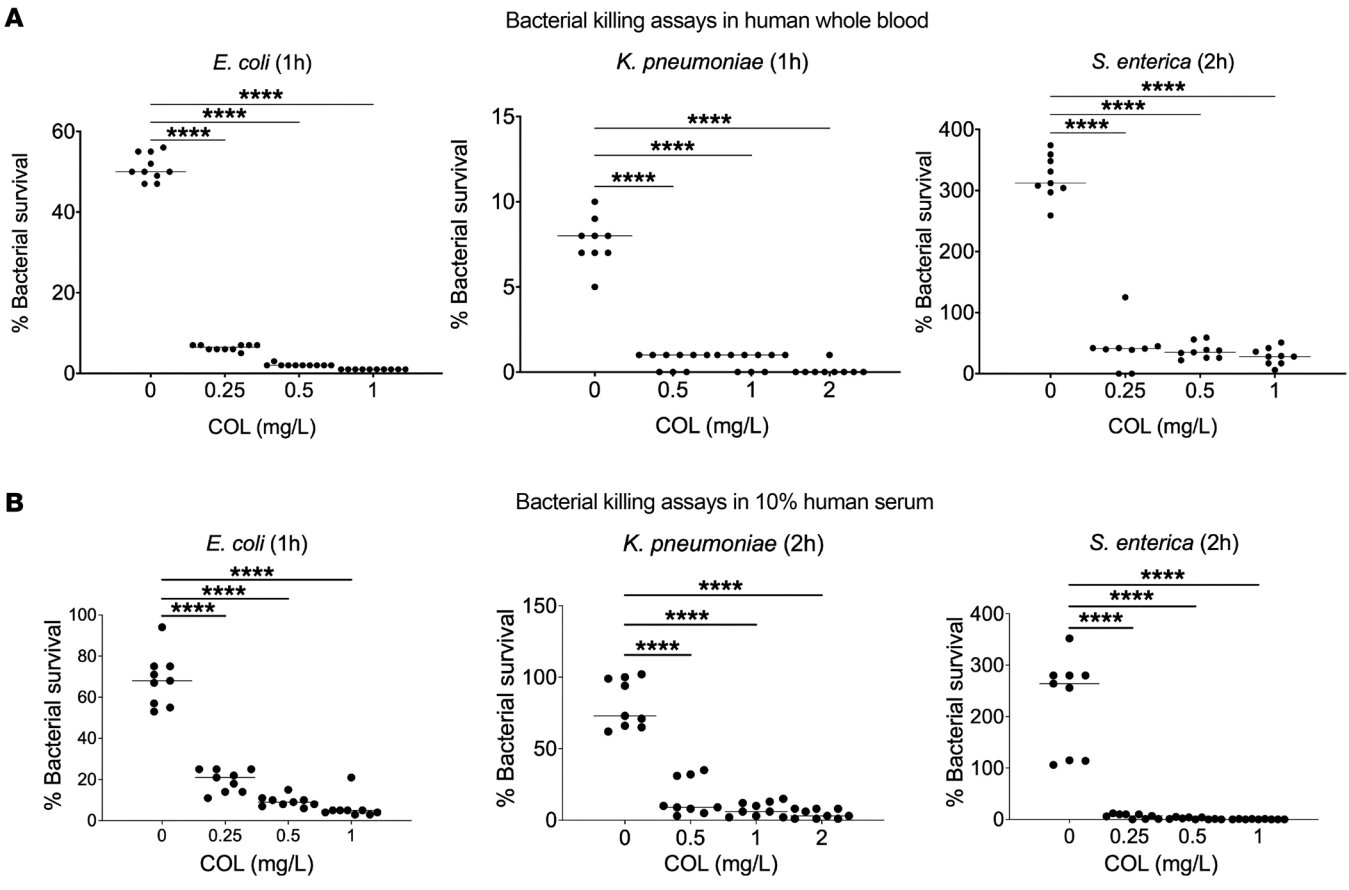
COL accelerates killing of *mcr-1^+^* Gram-negative bacteria in human blood and serum. (**A**) Bacterial survival in freshly isolated human whole blood with or without COL at concentrations representing ¼×, ½×, and 1× MIC for each *mcr-1^+^* bacterial strain. (**B**) Bacterial survival in 10% human serum with or without COL at concentrations representing ¼×, ½×, and 1× MIC for each *mcr-1^+^* bacterial strain. Data are represented as percentage viable CFU versus initial inoculum and mean ± SEM from a combination of 3 experiments performed in triplicate using blood or serum from 3 different donors. ****P ≤ 0.0001 by 1-way ANOVA.

We next explored whether the activity of COL against leading *mcr-1^+^* Gram-negative bacterial pathogens uncovered in the physiological RPMI(10%LB) medium held true in more complex infection-relevant matrices of human blood. Whole blood was freshly collected from normal human volunteers and inoculated with *mcr-1^+^* strains of *E*. *coli*, *K*. *pneumoniae*, or *S*. *enterica*, with COL added at 0.25 times, 0.5 times, and 1 time the respective MIC identified in RPMI(10%LB), and bacterial CFUs were enumerated at 1 or 2 hours for comparison with the initial inoculum. A significant enhancement of killing of both *mcr-1^+^* *E*. *coli* and *K*. *pneumoniae* was observed upon addition of COL, even at the lowest 0.25× MIC concentration at 1 hour (Figure 2A). While the *mcr-1^+^* *S*. *enterica* proliferated in human whole blood in the absence of antibiotic, addition of even 0.25× MIC COL significantly reduced recovered CFU over a 2-hour period (Figure 2A). A critical element of innate defense against Gram-negative bacteria in blood is the lytic action of serum complement, and recent work indicates that complement can serve to sensitize these bacteria to antibiotics including azithromycin, nisin, avibactam, and vancomycin that are normally considered ineffective based solely on standard AST in bacteriological media (24, 33, 38, 39). Paralleling our findings in whole blood, we identified clear and significant synergy of COL, added at as low as 0.25× MIC, to potentiate killing of *mcr-1^+^* *E*. *coli*, *K*. *pneumoniae*, and *S*. *enterica* in 10% human serum (Figure 2B). Synergy of COL with human serum was extended to the larger panel of 12 Gram-negative bacteria harboring *mcr* plasmids by addition of 10% serum to a modified checkerboard MIC assay performed in standard CA-MHB bacteriological medium. For 10 of these isolates, a significantly reduced COL MIC was calculated in the presence of 10% serum (Table 2); two strains could not be assessed, as they failed to grow in the 10% serum alone. For all strains, 10% heat-inactivated serum did not lower the COL MIC, showing that functional complement was required. The MIC of COL dropped to ≤2 mg/L, the published EUCAST clinical breakpoint for colistin for Enterobacterales, when 10% human serum was added to the standard testing medium.

### COL promotes C3 deposition on the mcr-1^+^ Gram-negative bacterial surface.

**Figure 3:**
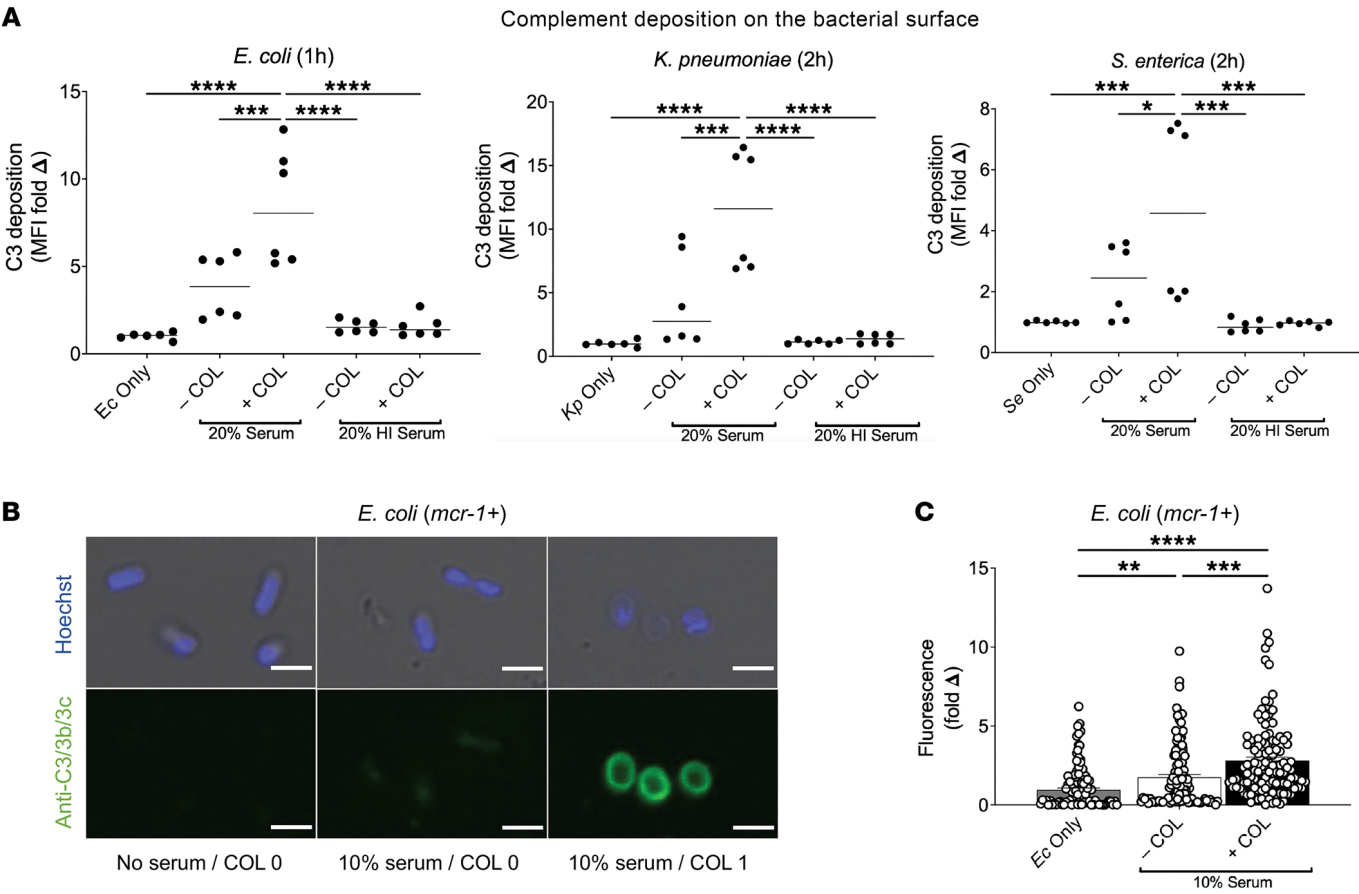
COL promotes C3 deposition on the *mcr-1^+^* Gram-negative bacterial surface. (**A**) C3 protein deposition on the surface of *E*. *coli*, *K*. *pneumoniae*, and *S*. *enterica* as detected by flow cytometry. Median fluorescence intensity (MFI) of bacteria-bound C3 protein shown as fold change versus untreated control bacteria of each species. Flow cytometry data are representative of 2 independent experiments conducted in triplicate; 10,000 cells were counted per experimental replicate, and analyses were performed using serum from 2 different donors. (**B**) Confocal microscopy images (average-intensity *Z* projections) of *E*. *coli* in the presence or absence of serum and presence or absence of 1 μg/mL COL; scale bars: 2 μm. DNA staining by Hoechst dye (blue); bound C3 protein detected using an anti-C3 antibody and fluorescent secondary antibody (Alexa Fluor 488, green). (**C**) Quantification of C3 fluorescence signal (average-intensity *Z* projections) from confocal microscopy shown as fold change versus untreated control bacteria. Bar graph generated from unbiased analysis of multiple random microscopy fields with more than 100 cells counted per condition. *P ≤ 0.05, **P ≤ 0.01, ***P ≤ 0.001, ****P ≤ 0.0001 by 2-way ANOVA.

Given their bactericidal synergy, we investigated the impact of the sequence of exposure to COL (0.25 μg/mL) and 10% human serum on killing of *mcr-1^+^* *E*. *coli* (Table 3). *E*. *coli* initially treated with COL for 30 minutes, followed by PBS washing, then subsequent exposure to 10% human serum for 30 minutes were rapidly killed (0% survival), compared with bacteria first exposed to 10% human serum for 30 minutes, followed by PBS washing, then subsequent exposure to COL for 30 minutes (52.2% bacterial survival). The bactericidal effect was abolished for both combinations when the serum complement was heat inactivated. To probe the mechanism by which COL may sensitize *mcr-1^+^* Gram-negative bacteria to serum killing, we hypothesized that sub-bactericidal COL could promote increased complement deposition and activation on the bacterial surface. All 3 major pathways of complement activation — the classical, lectin, and alternative pathways — converge at the point of C3 activation (40, 41) and the deposition and activation of C3 on the bacterial cell surface. The bound C3b can be recognized by cognate complement receptors on neutrophils and macrophages for phagocytic uptake (42), or initiate downstream complement cascades culminating in formation of the lytic membrane attack complex (MAC) (43). We assessed C3 binding to the bacterial cell surface using a C3/C3b/C3c antibody by flow cytometry and immunofluorescence microscopy in the presence and absence of COL. At 1× MIC (1 or 2 mg/L) in 20% human serum, COL significantly increased C3 cell surface deposition approximately ≥2-fold on *mcr-1^+^ E*. *coli*, *K*. *pneumoniae*, and *S*. *enterica* compared with bacteria in 20% human serum alone (Figure 3A). By quantitative fluorescence microscopy, *E*. *coli* exposed to 10% human serum had a 1.7-fold increase in C3 protein binding, while *E*. *coli* treated with serum plus COL (1× MIC) had a 2.8-fold increase in C3 binding, compared with untreated control (Figure 3, B and C). Flow cytometry and microscopy differences between COL-treated and -untreated *E*. *coli* were not seen in control assays performed in 10% heat-inactivated human serum.

### COL monotherapy reduces bacterial load and increases survival in a murine model of mcr-1^+^ E. coli bacteremia.

**Figure 4:**
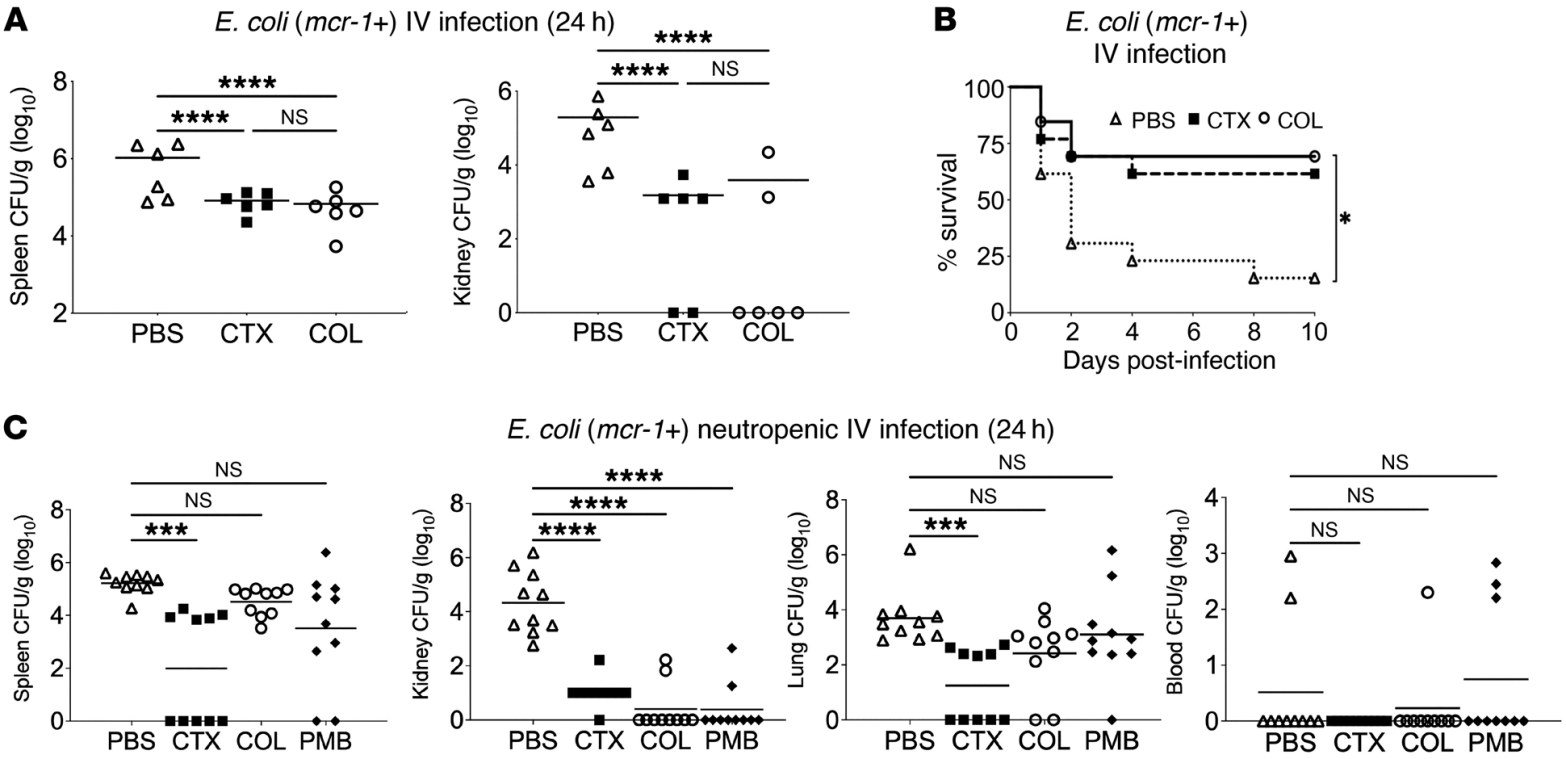
Impacts of polymyxin monotherapy on *mcr-1^+^* *E*. *coli* bacteremia outcomes in normal and neutropenic mice. (**A**) C57BL/6J mice were infected i.v. with 2 × 10^7^ CFU of *E*. *coli* (*mcr-1^+^*) and treated subcutaneously with PBS (100 μL, triangles), CTX (50 mg/kg, squares), or COL (20 mg/kg, circles) every 12 hours for 2 doses (*n =* 6 per group). Bacterial loads were recovered from spleen or kidney at 24 hours and are plotted as CFU/g organ tissue. (**B**) Survival of C57BL/6J mice infected i.v. with 1 × 10^9^ CFU of *E*. *coli* (*mcr-1*) and treated subcutaneously with a single dose of PBS (100 μL, triangles), CTX (50 mg/kg, squares), or COL (20 mg/kg, circles) 1 hour after infection (*n =* 13 per group). *P ≤ 0.05, ****P ≤ 0.0001 by 1-way ANOVA for CFU studies and log-rank test for survival. (**C**) Neutrophil-depleted C57BL/6J mice were infected with 2.5 × 10^5^ CFU of *E*. *coli* (*mcr-1*) and treated subcutaneously with PBS (100 μL, triangles), CTX (50 mg/kg, squares), COL (20 mg/kg, circles), or PMB (20 mg/kg, diamonds) every 12 hours for 2 doses (*n =* 10 per group). Bacterial loads were recovered from spleen, kidney, lung, or blood at 24 hours and are plotted as CFU/g organ tissue. ***P ≤ 0.001, ****P ≤ 0.0001 by 1-way ANOVA.

AST performed in standard CA-MHB bacteriological medium identifies *mcr-1^+^* bacteria as resistant to the action of COL, while comparable testing in the more physiological tissue culture–type medium RPMI(10%LB) or in the presence of human blood or serum reveals significant “hidden” bactericidal potential of the antibiotic against these same bacteria. To ascertain the in vivo relevance of these findings, we infected C57BL/6J mice i.v. with 2 × 10^7^ CFU of an *mcr-1^+^ E*. *coli* strain identified as resistant to COL in CA-MHB but sensitive to COL in RPMI(10%LB), blood, or serum. Groups of mice were then treated at 1 hour and 12 hours after infection with either COL (20 mg/kg/dose), PBS negative control, or, as a positive control, 50 mg/kg/dose of ceftriaxone (CTX), a commonly prescribed third-generation cephalosporin antibiotic to which this *E*. *coli* strain is sensitive in standard CA-MHB AST. Bacterial CFU recovered from the spleen of mice at 24 hours showed an approximately 15.5-fold reduction with COL treatment and an approximately 12.8-fold reduction with CTX treatment compared with PBS control (Figure 4A). Simultaneous examination of bacterial burden in the kidneys found significant reductions with both antibiotics, with 4 of 6 mice treated with COL and 2 of 6 mice treated with CTX below the detection limit for CFU recovery (Figure 4A). We next challenged groups of mice i.v. with a higher lethal dose of 1 × 10^9^ CFU of the same *mcr-1^+^ E*. *coli* strain, followed by a single dose 1 hour after infection of the same antibiotic treatments (20 mg/kg COL, 50 mg/kg CTX, or PBS control). Whereas 85% of the PBS control mice died of infection in the 10-day observation period, mice receiving COL (69% survival) or CTX (61% survival) experienced significant protection from mortality (Figure 4B). Thus, even though the *mcr-1^+^ E*. *coli* exhibited COL resistance by standard MIC testing in CA-MHB, the cationic peptide antibiotic performed equivalently well to the standard-of-care cephalosporin CTX for treatment of systemic *E*. *coli* infection in vivo.

### Host defense sensitizing activities of polymyxins against mcr-1^+^ E. coli depend in part on the presence and functionality of neutrophils.

To assess the impact of neutrophil depletion on bacterial clearance, we used a neutropenic mouse i.v. infection model (Figure 4C). Neutropenic mice were infected with 2.5 × 10^6^ CFU of the *mcr-1^+^* *E*. *coli* strain and subsequently treated with COL (20 mg/kg), PMB (20 mg/kg), CTX (50 mg/kg) as a positive control, or a PBS negative control at 1 hour and 13 hours after infection. Analysis of bacterial CFUs recovered from the lung and spleen of neutropenic mice at 24 hours after infection showed a trend toward reduction in CFUs recovered for mice treated with the polymyxins COL (2.4 and 4.5 log CFU/g) and PMB (3.1 and 3.5 log CFU/g) compared with the PBS control (3.7 and 5.2 log CFU/g) (Figure 4C), but these differences did not reach statistical significance. Treatment with the positive control cephalosporin CTX significantly reduced bacterial load in the lung (1.2 vs. 3.7 log CFU/g) and spleen (2.0 vs. 5.2 log CFU/g) compared with the PBS control. Bacterial recovery from the blood of neutropenic mice treated with COL, PMB, and CTX at 24 hours was limited, consistent with a transient bacteremia and endogenous seeding (Figure 4C). However, mirroring the findings in the immunocompetent murine model, a substantial decrease in bacterial burden was observed in the kidneys of neutropenic mice treated with the polymyxins COL and PMB, as well as the cephalosporin CTX, compared with the PBS control (Figure 4C). This finding holds particular significance in the kidneys, where polymyxins are known to attain high tissue concentrations (44–47). Neutrophil-mediated phagocytosis, a critical host defense mechanism, requires the opsonization of the bacterial surface, a process facilitated by the binding of serum complement proteins (such as C3b) and antibodies. Findings from lung and spleen in the neutropenic murine model, coupled with the COL sensitization to serum complement deposition and killing assays, indicate that functional neutrophil activity is important for the full impact of in vivo polymyxin host defense sensitizing activities against *mcr-1^+^* *E*. *coli* to be seen in the murine model.

### Increased susceptibility in physiological medium is seen in a subset of polymyxin-resistant Gram-negative bacterial strains lacking mcr genes.

Fifteen strains of polymyxin-resistant Gram-negative bacterial strains lacking *mcr* genes were collected from the Centers for Disease Control and Prevention (CDC) and US Food and Drug Administration (FDA) Antimicrobial Resistance Isolate Bank (Supplemental Table 1). Notably, a significant multifold reduction in COL and PMB MICs to ≤2 mg/L was observed in 7 of 15 strains when tested in RPMI(10%LB) compared with CA-MHB. These strains included 3 *K*. *pneumoniae*, 1 *Enterobacter cloacae*, 1 *Pseudomonas aeruginosa*, and 2 *Acinetobacter baumannii*. Conversely, no differences in MIC values in RPMI(10%LB) versus CA-MHB were seen among bacteria known to be intrinsically resistant to polymyxins, such as *Providencia stuartii*, *Serratia marcescens*, *Morganella morganii*, and *Proteus mirabilis*. Analysis was performed on the genome sequences of these 15 strains (see Methods and Supplemental Data Files 1–3) to identify the presence of mutations in any of 29 genes known to be involved in acquired polymyxin resistance (10, 48). Among the strains exhibiting differences in polymyxin MICs in RPMI(10%LB), the three *K*. *pneumoniae* strains harbored a R256G polymorphism in *pmrAB* linked to COL resistance (9, 49); no specific known polymyxin resistance–associated mutations were identified for the *E*. *cloacae*, *P*. *aeruginosa*, or *A*. *baumannii* isolates susceptible to COL and PMB in RPMI(10%LB).

## Discussion

The experiments we present highlighting the antibiotic action of COL against *mcr-1^+^* Gram-negative bacteria are straightforward, encompassing MIC testing, kinetic killing assays, synergy testing, and in vivo treatment in an animal model, yet their overarching medical implications are acute and significant. In the present era of expanding MDR and extensively drug-resistant (XDR) Gram-negative bacterial infections, polymyxins (COL and PMB) have reemerged as a critical and last-line antimicrobial therapy. The recent identification of *mcr-1*, the plasmid-borne and horizontally transmissible COL resistance gene, and its spread to superbugs resistant to nearly all antibiotics in our existing antimicrobial arsenal have raised a global public health alarm. If a clinical isolate presents a COL MIC in AST testing above the published CLSI or EUCAST cutoffs for susceptibility, or if *mcr-1* is detected through FDA-approved molecular-based diagnostics such as the BioFire (BioFire Diagnostics) or Verigene (Diasorin) blood culture panels (50–52), then clinicians, including infectious disease specialists, undoubtedly refrain from using COL in the treatment regimen for the infected patient. This logic and calculus, however, is firmly rooted in information provided by AST performed in nonphysiological bacteriological growth media.

Despite the lack of appreciable therapeutic antibacterial activity in the standard AST paradigm, our study revealed striking bactericidal activity of COL against several clinically important *mcr^+^* Gram-negative pathogens when testing was performed in the supplemented tissue culture medium, RPMI(10%LB). COL activity against *mcr-1^+^* *E*. *coli* required the crucial physiological buffer bicarbonate and paralleled results obtained ex vivo in human whole blood and in vivo in a murine model of bacteremia. Indeed, COL was noninferior to the comparator CTX (identified to have a therapeutic MIC in CA-MHB) in murine survival and bacterial burden from harvested organs. Furthermore, insights garnered from a neutropenic murine model, coupled with serum complement killing assays, imply that functional neutrophil activity (opsonophagocytosis) may play a crucial role in the in vivo polymyxin host defense sensitizing activities against *mcr-1^+^* *E*. *coli*.

In the 1970s, decades before the discovery of *mcr-1*, a series of classical studies found additive or synergistic effects of PMB and human serum against Gram-negative pathogens including *E*. *coli*, *Serratia marcescens*, and *Salmonella* Typhimurium whether or not the strain exhibited intrinsic (chromosomal) resistance to polymyxins (53–55). We found that COL strongly potentiated serum killing of *mcr^+^* *E*. *coli*, *K*. *pneumoniae*, and *S*. *enterica* and that this effect was eliminated by heat inactivation of complement, a finding corroborated by flow cytometry and fluorescence microscopy demonstration of increased serum complement binding to bacterial surface in the presence of COL. Serum typically constitutes 55% of human blood volume, and with just 10% serum supplementation we found marked sensitization of *mcr^+^* bacteria at COL concentrations (0.25 or 0.5 mg/L) well below the EUCAST clinical breakpoint for susceptibility (≤2 mg/L). Indeed, addition of 10% serum allowed COL to kill *mcr^+^* strains in both the physiological medium RPMI(10%LB) and standard CA-MHB bacteriological medium in our MIC assays (Tables 1 and 2), suggesting that a simple modification to standard clinical microbiology laboratory workflows is at hand to discover these hidden and potentially clinically impactful antibiotic activities.

Furthermore, the host biological milieu has been shown to modulate bacterial growth, gene expression, and essential gene patterns of diverse pathogens, influencing their virulence and antimicrobial susceptibility. For instance, transcriptional profiling of MDR *P*. *aeruginosa* cultivated in host-mimicking tissue culture medium (RPMI 1640) versus standard bacteriological culture medium (CA-MHB) uncovered the dysregulation of several resistome genes underpinning antimicrobial susceptibility (56). A notable finding was the marked reduction in the expression of the *arn* operon (*arnBCADTEF*), which mediates resistance to cationic antimicrobials such as polymyxins through the modification of lipid A on LPS with 4-aminoarabinose, showing a 17- to 40-fold decrease in host-mimicking medium compared with CA-MHB (56). Additionally, the expression of regulators of the *arn* operon (*phoPQ*) and various susceptibility determinants (*parRS*, *pmrAB*, *galU*, *pyrU*, *pyrD*, *lptC*) known to contribute to PMB resistance induction was downregulated, whereas genes associated with LPS production were upregulated, in the supplemented tissue culture medium. Aligning with these observations, our investigation also demonstrated a marked reduction in *mcr-1* expression via quantitative RT-PCR in *E*. *coli* cultured in host-mimicking medium, which correlated with an increase in susceptibility and NPN permeabilization to COL.

We further observed increased susceptibility among a number of polymyxin-resistant Gram-negative bacteria that lack *mcr* genes when they are tested in the more physiological RPMI(10%LB) medium compared with the standard bacteriological testing medium CA-MHB, including *K*. *pneumoniae*, *E*. *cloacae*, *P*. *aeruginosa*, and *A*. *baumannii*. Of particular interest, Panta and Doerrler recently reported in vitro findings that alkaline pH or bicarbonate increased colistin activity against several COL-resistant Gram-negative bacterial species that likewise lack *mcr*, including *E*. *coli* WD102, *K*. *pneumoniae*, *Vibrio* *cholerae*, *Burkholderia* *thailandensis*, and *S*. *marcescens* (57). In addition, reduction of oxygen or glucose supplementation counteracted these effects by mitigating cytoplasmic pH changes.

As familiar therapeutic alternatives dwindle to a precarious few for established or emerging bacterial pathogens, including several MDR Gram-negative species of urgent concern, it is incumbent upon clinical microbiologists, infectious disease researchers, and clinicians to ensure that approved antibiotic agents are evaluated in the most comprehensive and holistic manner. Upon testing of *mcr^+^* strains of the Gram-negative pathogens *E*. *coli*, *K*. *pneumoniae*, and *S*. *enterica* in physiological medium and/or the presence of human serum, continued susceptibility to COL was apparent at drug levels readily attained with standard dosing and validated by potent COL killing of each *mcr^+^* pathogen in freshly isolated human blood and *mcr^+^* *E*. *coli* in a murine bacteremia model. We hope that these observations can inspire careful clinical trial design to determine whether continued COL administration can contribute to successful therapeutic regimens in serious human *mcr^+^* Gram-negative bacterial infections, and that similar analyses can be applied to other drug and pathogen combinations to ensure that familiar antibiotics are not being prematurely declared obsolete.

## Methods

### Sex as a biological variable.

All animal experiments were conducted using 8- to 10-week-old female C57BL/6J mice. Female mice were selected to minimize inter-animal variability related to sex hormones and aggression, which can occur more frequently among group-housed male mice. While only one sex was used, the bacterial strains studied, and the antimicrobial mechanisms being evaluated, are not known to exhibit sex-specific effects in mice. As such, the findings are expected to be broadly relevant to both sexes. Sex as a biological variable was not directly investigated in this study.

### Bacterial strains, media, and antibiotics.

Twelve Enterobacteriaceae strains harboring *mcr-1* to *mcr-4* chosen from the Isolates with New or Novel Antibiotic Resistance Panel (CDC and FDA Antimicrobial Resistance Isolate Bank) (58) were investigated for susceptibility to the cationic peptide antibiotics COL and PMB. Three *mcr-1^+^* strains, *E*. *coli* (AR Bank #0494), *K*. *pneumoniae* (AR Bank #0497), and *S*. *enterica* (AR Bank #0496), were used in subsequent experiments. Isolates were stored in Luria-Bertani broth  plus  50% glycerol at –80°C until use. COL, PMB, and CTX were purchased from Sigma-Aldrich. The bacteriological medium Mueller-Hinton broth (Difco) was supplemented with 20–25 mg/L Ca^2+^ and 10–12.5 mg/L Mg^2+^ (CA-MHB). The tissue culture medium Roswell Park Memorial Institute (RPMI) 1640 (Thermo Fisher Scientific) was supplemented with 10% Luria-Bertani (LB) broth (Hardy Diagnostics), yielding RPMI (10%LB).

### Antibiotic susceptibility assay.

MIC assays of COL and PMB were performed per CLSI and EUCAST guidelines using the broth microdilution methodology with the recommended standard medium (CA-MHB), the alternative cell culture medium containing bicarbonate buffer [RPMI(10%LB)], and a final bacterial concentration of 5 × 10^5^ CFU/mL. Further assays were performed in the presence or absence of 10% human serum or 10% heat-inactivated human serum. MICs were determined based on visual turbidity and absorbance (OD_600_) after 20–22 hours of incubation at 37°C.

### Kinetic killing assay.

*E*. *coli*, *K*. *pneumoniae*, and *S*. *enterica* were grown overnight in LB broth, washed twice, and diluted in CA-MHB, CA-MHB plus COL (1× MIC), RPMI(10%LB), and RPMI(10%LB) plus COL (1× MIC) to 5 × 10^5^ CFU/mL using a 96-well round-bottom plate, in triplicate wells at a final volume of 100 μL/well, and incubated at 37°C with shaking for 24 hours. Samples were collected at 0, 2, 4, 8, and 24 hours of incubation, serially diluted in sterile PBS, and plated on LB agar for CFU enumeration. Bactericidal activity was defined as a reduction in viable bacteria by ≥3 log_10_ CFU/mL at 24 hours compared with the starting inoculum.

### Bicarbonate supplementation assay.

Overnight bacterial cultures (*E*. *coli*, *K*. *pneumoniae*, *S*. *enterica*) grown in LB broth were washed twice and diluted to 5 × 10^5^ CFU/mL in CA-MHB (with 100 mM Tris) or RPMI(10%LB), with the pH adjusted to approximately 7.4 before the addition of 0, 10, or 25 mM of NaHCO_3_ (spanning physiological NaHCO_3_ concentrations seen in humans). Then 0, 1, or 2 μg/mL of COL was added to a final volume of 100 μL, with the assay performed in triplicate wells of a 96-well round-bottom plate. Plates were incubated at 37°C with shaking for 24 hours before being serially diluted in sterile PBS and plated on LB agar for CFU enumeration. Surviving bacteria were expressed as log_10_ CFU/mL.

### NPN outer membrane permeabilization assay.

Overnight cultures of *E*. *coli* grown in LB broth at 37°C in a shaking incubator were washed twice with PBS via centrifugation at 5,000*g* and then resuspended in 10 mL of CA-MHB, CA-MHB plus COL (1 μg/mL), RPMI(10%LB), or RPMI(10%LB) plus COL (1 μg/mL) at OD_600_ = 0.4. Cultures were incubated for 2 hours at 37°C in a shaker, centrifuged at 4,000 rpm for 5 minutes, and resuspended in 2 mL of 10 mM Tris buffer (pH 8.0). The concentrated 2 mL cultures were used to prepare 4 mL bacterial stocks in 10 mM Tris at OD_600_ = 0.4. Assays were performed at a final volume of 200 μL in triplicate and using a 96-well flat-bottom plate. The 4 conditions tested were: (a) bacteria (100 μL) + 40 μM 1-*N*-phenylnaphthylamine (NPN) (50 μL) + 10 mM Tris (50 μL); (b) bacteria (100 μL) + 40 μM NPN (50 μL) + 10 mM EDTA (50 μL); (c) bacteria (100 μL) + 10 mM EDTA (50 μL) + 10 mM Tris (50 μL); and (d) 10 mM EDTA (50 μL) + 40 μM NPN (50 μL) + 10 mM Tris (100 μL). After addition of all components and mixing, fluorescence was immediately read at an excitation and emission of 250 nm and 420 nm, respectively. To obtain the NPN fluorescence signal measured, conditions 3 and 4 (background) were subtracted from conditions 1 and 2, respectively. After subtraction of background, the percentage NPN outer membrane permeabilization in the presence of EDTA was determined by dividing the measured NPN fluorescence signal of condition 1 (bacteria + NPN) from condition 2 (bacteria + NPN + EDTA) (24, 59).

### RNA extraction and quantitative RT-PCR.

*E*. *coli* were grown overnight (18 hours) in the following conditions: CA-MHB, CA-MHB + COL (0.25 μg/mL), CA-MHB + 0.02% l-arabinose (a known inducer of *mcr-1* expression), RPMI(10%LB), RPMI(10%LB) + COL (0.25 μg/mL), and RPMI(10%LB) + 0.02% l-arabinose; washed 3 times with PBS via centrifugation at 5,000*g* for 5 minutes; and then resuspended in the corresponding media to OD_600_ = 0.4. Total RNA was extracted from the washed overnight cultures using the ZymoBIOMICS DNA/RNA Miniprep Kit (Zymo Research). Bacteria were mechanically disrupted and homogenized in a high-speed bead beater at maximum speed for 5 minutes. RNA was treated with DNase (Turbo DNase, Ambion), and 1 μg total RNA was reverse-transcribed to cDNA (iScript, Bio-Rad). Real-time PCR was performed using PerfeCTa SYBR Green Supermix (Quantabio) with the following primer sets at 1 μM final concentration: *mcr-1* forward 5′-TGGCGTTCAGCAGTCATTAT-3′ and *mcr-1* reverse 5′-AGCTTACCCACCGAGTAGAT-3′; 16S forward 5′-CATTGACGTTACCCGCAGAA-3′ and 16S reverse 5′-CGCTTTACGCCCAGTAATTCC-3′. Data were normalized to the housekeeping gene 16S, and relative expression in CA-MHB was compared with that in RPMI(10%LB) by the ΔΔCt method using mean Ct value (60).

### Whole-blood killing.

Stationary-phase bacteria (*E*. *coli*, *K*. *pneumoniae*, and *S*. *enterica*) were washed twice, diluted to an inoculum of 1 × 10^6^ to 2 × 10^6^ CFU in 50 μL PBS, and mixed with 400 μL heparinized human whole blood and 50 μL PBS with or without COL (at ¼× MIC, ½× MIC, or 1× MIC identified in supplemented RPMI) in siliconized tubes. Tubes were incubated at 37°C and rotated for 1 and 2 hours. After incubation, the infected blood was serially diluted using sterile PBS and 0.025% Triton X-100 and plated on LB agar plates. Percentage bacterial survival was defined as the number of CFUs enumerated divided by the initial bacterial inoculum × 100%.

### Serum complement killing.

Human serum was pooled from 3 healthy donors, stored as small aliquots at –80°C for no longer than 3 months, and thawed on the day of the experiment (kept at 4°C for about 1 hour before use). Heat-inactivated normal human serum (serum heated to 56°C for 30 minutes before use) served as a control. Overnight bacterial cultures (*E*. *coli*, *K*. *pneumoniae*, *S*. *enterica*) grown in LB broth were washed twice and diluted to approximately 2 × 10^8^ CFU/mL in RPMI 1640. To measure bacterial survival, 1 × 10^6^ to 2 × 10^6^ bacterial CFU in 20 μL was added to RPMI 1640 with or without 10% pooled human serum and varying concentrations of COL (0 mg/L, ¼× MIC, ½× MIC, or 1× MIC identified in RPMI) to a final volume of 200 μL in siliconized tubes rotated at 37°C. Samples were collected at 1 and 2 hours, serially diluted in PBS, and plated on LB agar for CFU enumeration. Percentage survival was defined as the number of CFUs enumerated divided by the initial bacterial inoculum × 100%.

### Complement deposition.

C3 deposition on the bacterial surface was determined as previously described (61, 62) with minor modifications. Overnight bacterial cultures (*E*. *coli*, *K*. *pneumoniae*, *S*. *enterica*) grown in LB broth were washed 3 times with PBS and resuspended in RPMI 1640. Next, 1 × 10^6^ to 2 × 10^6^ CFU of bacteria in 20 μL was added to RPMI 1640 containing no human serum, 20% human serum with or without 1× MIC of COL, or 20% heat-inactivated human serum with or without 1× MIC of COL to a final volume of 200 μL. Bacterial samples were then incubated at 37°C for 1 hour (*E*. *coli*) or 2 hours (*K*. *pneumoniae* and *S*. *enterica*). After incubation, bacteria were washed with PBS 3 times, stained with a 1:200 dilution of LIVE/DEAD Fixable Violet Dead Cell Stain (Invitrogen) in PBS, and then incubated for 30 minutes at room temperature. Afterward, bacteria were washed with PBS 3 times and fixed with 4% paraformaldehyde for 20 minutes at room temperature. Bacteria were then washed 3 times with PBS, incubated for 30 minutes at room temperature with blocking buffer (10% BSA plus 0.01% of NaN_3_ in PBS), pelleted at 5,000*g* for 10 minutes, then resuspended in 1:500 dilution of rabbit anti-C3/C3b/C3c primary antibody (Proteintech, 21337-1-Ab) with blocking buffer and incubated for 30 minutes at 4°C. Bacteria were then washed with PBS 3 times, resuspended in 1:5,000 dilution of Alexa Fluor 488–labeled goat anti-rabbit IgG secondary antibody (Invitrogen, A-11070) with blocking buffer, and incubated for 30 minutes at 4°C. Lastly, bacteria were washed with PBS 3 times before being resuspended in PBS and immediately analyzed using the BD FACSCanto II flow cytometer (BD Biosciences). Forward scatter and side scatter were used to exclude debris and aggregates, and 10,000 gated events were recorded for each sample. Data were then analyzed with FlowJo v10.2 software (FlowJo LLC) to identify the mean and median fluorescence intensity. Negative controls including bacteria without serum and bacteria stained with solely secondary antibody were used for setting gate boundaries. Additionally, heat-killed bacteria were used as a positive control for the dead cell stain. Each condition and controls were performed in triplicate, and all incubations were maintained in a light-free environment.

### Immunofluorescence.

For fluorescence microscopy, *E*. *coli* (*mcr-1*) were prepared and incubated at 37°C for 1 hour as described above. Bacteria were then washed 3 times with PBS and allowed to bind onto poly-d-lysine–coated coverslips for 1 hour (Corning). Next, coverslip-bound bacteria were fixed with 4% paraformaldehyde in PBS, and coverslips were blocked by incubation in blocking buffer (5% BSA plus 0.02% of NaN_3_ in PBS) for 30 minutes at room temperature and stained with 1:500 dilution of rabbit anti-C3/C3b/C3c primary antibody (Proteintech, 21337-1-Ab) for 1 hour at room temperature. Coverslips were washed 3 times in PBS, stained with 1:1,000 Alexa Fluor 488–labeled goat anti-rabbit IgG secondary antibody (Invitrogen, A-11070) with blocking buffer, and incubated for 1 hour at room temperature before being washed again 3 times with PBS. Coverslips were stained with 2 μM Hoechst dye (Thermo Fisher Scientific) and incubated for 15 minutes at room temperature, washed 3 times with PBS, and then mounted onto slides with ProLong Gold Antifade Mountant (Invitrogen). Images were acquired using a Leica SP8 Super Resolution Confocal microscope with ×1,000 final magnification. ImageJ (NIH) was used to construct *Z*-stacks utilizing 6 optical slices, and average-intensity projections were generated. An outline of 120 bacteria was traced in the *Z* projections, and the integrated density and cell area were recorded. Mean fluorescence of 4 surrounding background regions was also measured. The corrected total cellular fluorescence (CTCF) was calculated as CTCF = integrated density – (area of selected cell × mean fluorescence of background regions). The relative fold CTCF was calculated and reported as fold fluorescence in comparison with untreated *E*. *coli*.

### Serum and COL exposure assay.

Overnight cultures of *mcr-1^+^* *E*. *coli* grown in LB broth at 37°C in a shaking incubator were washed twice with PBS, and then diluted to 2 × 10^8^ CFU/mL in 3 mL of RPMI(10%LB) with 10% human serum (tube 1), COL (0.25 μg/mL) (tube 2), 10% heat-inactivated human serum (tube 3), or COL (0.25 μg/mL) (tube 4) in 5 mL round-bottom tubes. Cultures were incubated for 30 minutes at 37°C in a shaker. After incubation, 1 mL was removed from each tube for serial dilution using sterile PBS and plated on LB agar plates for bacterial enumeration (pre-exposure). Next, the remaining volume of 2 mL in each tube was washed twice with PBS, and then resuspended in 2 mL of RPMI(10%LB) with COL (0.25 μg/mL) (tube 1), 10% human serum (tube 2), COL (0.25 μg/mL) (tube 3), or 10% heat-inactivated human serum (tube 4). Resuspended cultures were then incubated for 30 minutes at 37°C in a shaker. After incubation, 1 mL was removed from each tube for serial dilution using sterile PBS, and plated on LB agar plates for bacterial enumeration (post-exposure). Percentage bacterial survival was defined as the number of CFU/mL enumerated post-exposure divided by the CFU/mL enumerated pre-exposure × 100%.

### Murine intravenous infection model.

The in vivo activity of COL and CTX against *mcr-1^+^* *E*. *coli* was evaluated using an immunocompetent murine intravenous challenge (63). Eight- to 10-week-old female C57BL/6J mice (The Jackson Laboratory) were infected i.v. via the right retro-orbital venous sinus with 2 × 10^7^ CFU of *E*. *coli* in 100 μL PBS for the bacterial burden study and treated with COL (20 mg/kg, subcutaneously [s.c.], *n =* 6), CTX (50 mg/kg, s.c., *n =* 6), or PBS (100 μL, s.c., *n =* 6) every 12 hours (at 1 and 13 hours after infection) for a total of 2 doses. Mice were euthanized 24 hours after infection by CO_2_ asphyxiation and cervical dislocation. Organs (right kidney and spleen) were aseptically collected, weighed, homogenized, serially diluted, and plated on LB agar for CFU enumeration after 24 hours of incubation at 37°C. To assess survival, all operations of infection were similar, except mice were infected i.v. with 1 × 10^9^ CFU of *E*. *coli* (*mcr-1*) in 100 μL PBS and treated with only 1 dose of COL (20 mg/kg, s.c., *n =* 13), CTX (50 mg/kg, s.c., *n =* 13), or PBS (100 μL, s.c., *n =* 13) 1 hour after infection. Survival was monitored every 12 hours until the endpoint of the experiment (defined as 10 days after infection), and date and time of death were recorded for each mouse.

### Neutropenic murine intravenous infection model.

Neutropenia was induced in 8- to 10-week-old female C57BL/6J mice (The Jackson Laboratory) by intraperitoneal treatment with anti–mouse Ly6G (250 μg/mouse on day 1, 100 μg/mouse on day 2). Peripheral blood was obtained from some mice 24 hours after treatment (*n =* 3) to confirm neutropenia via cell blood cell count with differential by hemocytometry and Wright-stained blood smears. Mice were infected with 2.5 × 10^6^ CFU of *E*. *coli* (*mcr-1*) in 100 μL PBS i.v. via the right retro-orbital venous sinus 1 hour after completion of anti–mouse Ly6G administration and treated with COL (20 mg/kg, s.c., *n =* 10), PMB (20 mg/kg, s.c., *n =* 10), CTX (50 mg/kg, s.c., *n =* 10), or PBS (100 μL, s.c., *n =* 10) every 12 hours (at 1 and 13 hours after infection) for a total of 2 doses. Mice were euthanized 24 hours after infection, and blood and organs (right kidney, spleen, lungs) were aseptically collected for serial dilution and plating on LB agar for CFU enumeration as described above.

### Statistics.

Statistical analyses were conducted using Prism 9.0 (GraphPad Software Inc.) on at least 3 independent experiments unless otherwise stated. Data are represented as the mean ± SEM where applicable, and *P* values less than 0.05 were regarded as statistically significant. Sample size and information about statistical tests are reported in Methods and the figure legends.

### Study approval.

Human blood and serum were obtained from healthy donors with informed consent under a simple phlebotomy IRB protocol, 131002, approved by the UCSD Human Research Protection Program. All murine infection and antibiotic treatment studies were conducted in compliance with federal Animal Welfare Act regulations and the UCSD Institutional Animal Care and Use Committee under approved protocol S00227M.

### Data availability.

The authors are committed to data transparency. The data used to generate figures can be accessed in the supplemental Supporting Data Values file and can be obtained upon request.

## Author contributions

MK contributed to research study design, experiments (MIC, checkerboards, kinetic killing, HCO_3_^–^ sensitization, membrane permeabilization, serum and whole-blood killing, complement studies, microscopy, mouse infections), data acquisition and analysis, figure generation, and manuscript writing. AMR contributed to research study design and conducted experiments (complement binding, flow cytometry, fluorescence microscopy), data acquisition and analysis, figure generation, and manuscript writing. AF conducted experiments (kinetic killing, HCO_3_^–^ sensitization, membrane permeabilization), data acquisition and analysis, figure generation, and manuscript writing. SD conducted experiments (kinetic killing, RNA extraction, quantitative RT-PCR, *mcr-1* PCR detection), data acquisition and analysis, figure generation, and manuscript writing. FA and SU conducted murine models of infection, data acquisition and analysis, and figure generation. JM conducted genome assembly and antibiotic resistance gene identification, data acquisition and analysis, and figure generation. SJ conducted experiments (MIC, kinetic killing), data acquisition and analysis, and figure generation. GB, V Nilsson, HS, and MC conducted preliminary experiments (MIC, whole-blood killing, RT-PCR analysis) and data acquisition and analysis. JBB, YL, TC, SJ, and GS contributed to research study design, data acquisition and analysis, and/or manuscript writing. AK, EB, and NC assisted FA with murine models of infection. V Nizet contributed to the overall research study design, data analysis, figure generation, and the writing and editing of the manuscript.

## Supplementary Material

Supplemental data

Supplemental Data File 2

Supplemental Data File 3

Supporting data values

## Figures and Tables

**Table 3:**
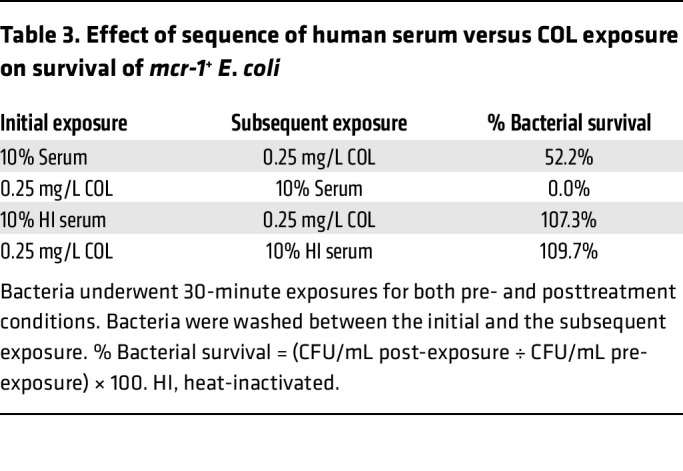
Effect of sequence of human serum versus COL exposure on survival of *mcr-1^+^ E*. *coli*

**Table 2:**
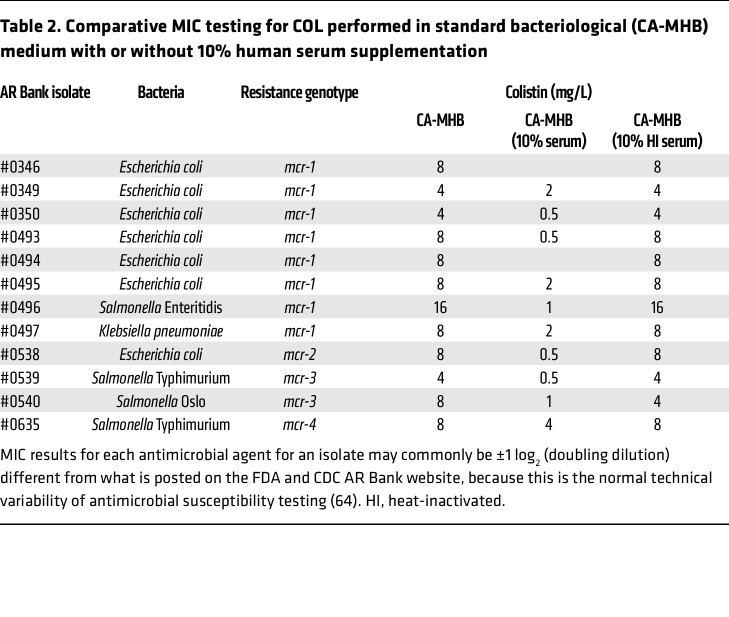
Comparative MIC testing for COL performed in standard bacteriological (CA-MHB) medium with or without 10% human serum supplementation

**Table 1:**
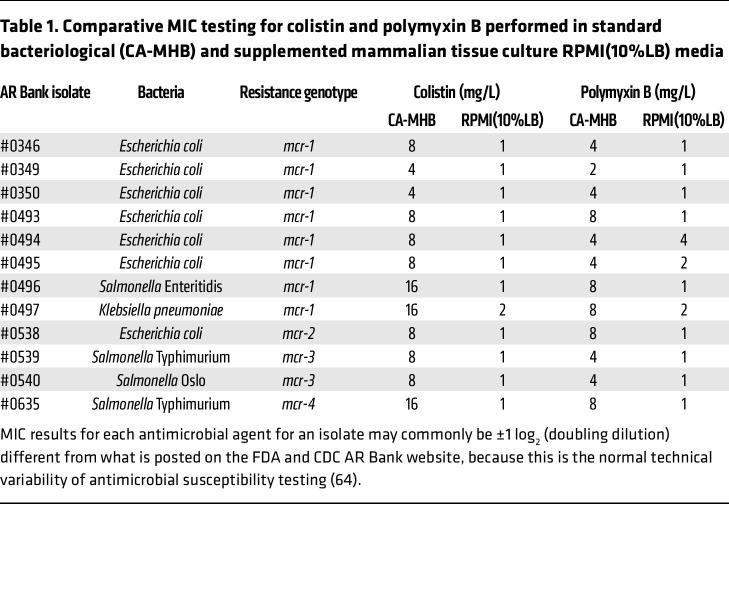
Comparative MIC testing for colistin and polymyxin B performed in standard bacteriological (CA-MHB) and supplemented mammalian tissue culture RPMI(10%LB) media

## Version 1 04/22/2025

In-Press Preview

## Version 2 06/16/2025

Electronic publication
