# Supplementary material for: Colistin exerts potent activity against mcr+ Enterobacteriaceae via synergistic interactions with the host defense
Source: J Clin Invest. 2025 Apr 22;135(12):e170690. doi: 10.1172/JCI170690 (PMC12165787; doi:10.1172/JCI170690)
Supplement: Supplemental data [file jci-135-170690-s218.pdf]

Supplementary Data File 1

Colistin Resistance Gene Identification

Whole-genome sequencing files were downloaded from the SRA for 15 strains of interest (**See Supplemental Table 1 Below**). Genomes were assembled using SPADES v3.13.0 and Unicycler v0.4.8 with default settings (1,2). Each assembled genome was annotated using Prokka v1.14.6 (3). A list of 29 genes involved in colistin resistance was compiled by reviewing literature (4,5). These genes were searched for in the genome annotations of each strain. In total 22 genes were identified across the 15 strains. Colistin resistance often arises from mutations in these genes of interest. Genes and mutations implicated in colistin resistance were searched for using the CARD v5.2.1 (**Details in Supplemental Data File 2, .xls format**) and ABRITAMR v1.0.13 databases (6,7). ABRITAMR screens for 196 genes and mutations implicated in colistin resistance that are species specific (**Details in Supplemental Data File 3, .xls format**) beyond the mcr genes including *arnC, basR, pmrB* and *crrB*. In total, only the three *Klebsiella* strains were found to have mutations implicated in resistance to colistin (R256G polymorphism in *PmrAB*).

1. Bankevich A, et al. SPAdes: a new genome assembly algorithm and its applications to single-cell sequencing. *J Comput Biol*. 2012;19(5):455-77.
2. Wick RR, et al. Unicycler: Resolving bacterial genome assemblies from short and long sequencing reads. *PLoS Comput Biol*. 2017;13(6):e1005595.
3. Seemann T. Prokka: rapid prokaryotic genome annotation. *Bioinformatics*. 2014; 15;30(14):2068-9.
4. Moffatt JH, et al. Mechanisms of polymyxin resistance. *Adv Exp Med Biol*. 2019;1145:55-71.
5. Olaitan AO, et al. Mechanisms of polymyxin resistance: acquired and intrinsic resistance in bacteria. *Front Microbiol*. 2014;5:643.
6. McArthur AG, et al. The comprehensive antibiotic resistance database. *Antimicrob Agents Chemother*. 2013; 57(7):3348-57.
7. Sherry NL, et al. An ISO-certified genomics workflow for identification and surveillance of antimicrobial resistance. *Nat Commun*. 2023; 14(1):60.

Supplementary Table 1. Comparative MIC testing for COL and PMB performed in standard bacteriological (CA-MHB) and supplemented mammalian tissue culture (RPMI (10%LB)) media.

AR Bank Isolate	Bacteria	Resistance Genotype	Colistin (mg/L)		Polymyxin B (mg/L)	
			CA-MHB	RPMI (10% LB)	CA-MHB	RPMI (10% LB)
#0026	*Providencia stuartii*	*aac(2')-Ia, catA3, QnrD1, tet(B)*	>16	>16	>16	>16
#0027	*Serratia marcescens*	*aac(6')-SERRA, SDEB, SMDA, SMDB, SME, SRT*	>16	>16	>16	>16
#0029	*Proteus mirabilis*	*tet(J)*	>16	>16	>16	>16
#0040	*Klebsiella pneumoniae*	*aac(3)-IIa, aac(6')-Ib-cr, aac(6')-II, aph(3')-Ia, aph(6)-Id, catB4, CTX-M-15, dfrA1, EMRD, KDEA, mph(A), Omp35, OmpK35, oqxA, oqxB25, OXA-1, qacEdelta1, SHV-11, strA, sul1, tet(A), tet(R), VIM-27*	8	1	8	2
#0046	*Klebsiella pneumoniae*	*aac(3)-IIa, aac(6')-Ib-cr, aac(6')-II, aph(3')-Ia, aph(6)-Id, catB4, CTX-M-15, dfrA1, EMRD, KDEA, mph(A), Omp35, OmpK35, oqxA, oqxB25, OXA-1, qacEdelta1, SHV-11, strA, sul1, tet(A), tet(R), VIM-27*	8	2	4	2
#0047	*Klebsiella pneumoniae*	*aac(6')-Ib, aadA2, catA1, dfrA12, EMRD, KDEA, mph(A), Omp35, OmpK35, oqxA, oqxB, qacEdelta1, sul1, TEM-1A, tet(D)*	>16	2	>16	2
#0057	*Morganella morganii*	*aac(3)-IIa, aac(6')-Ib-cr5, aadA2, aadA5, aph(3')-Ia, aph(3')-Ib, aph(6)-Id, ble-MBL, catA1, catB4, CTX-M-15, dfrA12, dfrA17, DHA-13, NDM-1, OXA-1, qacEdelta1, sul1, sul2, TEM-1, tet(B)*	>16	>16	>16	>16
#0121	*Serratia marcescens*	*SME-3*	>16	>16	>16	>16
#0133	*Morganella morganii*	*aadA1, dfrA1, dfrA14, KPC-2, tet(D)*	>16	>16	>16	>16
#0155	*Proteus mirabilis*	*aadA1, aadA5, dfrA1, dfrA17, KPC-6, sul1, sul2, tet(J)*	>16	>16	>16	>16
#0156	*Proteus mirabilis*	*cmlA1, dfrA1, dfrA14, KPC-2, OXA-10, sul1, sul2, tet(D), tet(J)*	>16	>16	>16	>16
#0163	*Enterobacter cloacae*	*aac(3)-IIa, ACT-7, CTX-M-15, dfrA14, KPC-2, OXA-1, strA, strB, sul2, TEM-1B*	16	1	16	1

#0239	*Pseudomonas aeruginosa*	*aac(6')-IB3, aac(6')-IIa, aac(6')-Il, ant(2")-Ia, aph(3')-Ia, aph(3')-IIb, aph(6)-Id, bcr1, cmlA5, dfrB5, floR2, fosA, GES-1, mexA, mexE, OXA-10, OXA-395, PDC-36, strA, sul1, tet(G),* ***VIM-11***	8	2	4	2
#0288	*Acinetobacter baumannii*	*aac(6')-Ib-AKT, aadA1, ABAF, ADC-30, ADEC, ant(3")-IIa, aph(6)-Id, armA, catB8, mph(E), msr(E), OXA-23, OXA-66, qacEdelta1, strA, sul1, tet(B)*	>16	1	>16	1
#0303	*Acinetobacter baumannii*	*aac(6')-Ib-AKT, aadA1, ABAF, ADC-162, ADEC, aph(3')-Ia, aph(6)-Id, aphA6, armA, catB8, mph(E), msr(E), OXA-23, OXA-66, qacEdelta1, strA, sul1, sul2, tet(B)*	>16	2	>16	1

Note: MIC results for each antimicrobial agent for an isolate may commonly be ± 1 log2 (doubling dilution) different than what is posted on the FDA & CDC AR Bank website because this is the normal technical variability of antimicrobial susceptibility testing (see J. H. Jorgensen. 1993. J Clin Microbiol. Vol 31[11]: 2841-2844).
